# Traffic air pollution and mortality from cardiovascular disease and all causes: a Danish cohort study

**DOI:** 10.1186/1476-069X-11-60

**Published:** 2012-09-05

**Authors:** Ole Raaschou-Nielsen, Zorana Jovanovic Andersen, Steen Solvang Jensen, Matthias Ketzel, Mette Sørensen, Johnni Hansen, Steffen Loft, Anne Tjønneland, Kim Overvad

**Affiliations:** 1 Danish Cancer Society Research Center, Copenhagen, Denmark; 2 Department of Public Health, Center for Epidemiology and Screening, University of Copenhagen, Copenhagen, Denmark; 3 Department of Environmental Science, Aarhus University, Roskilde, Denmark; 4 Department of Public Health, Section of Environmental Health, University of Copenhagen, Copenhagen, Denmark; 5 Department of Epidemiology, School of Public Health, Aarhus University, Aarhus, Denmark

**Keywords:** Traffic, Air pollution, Cardiovascular mortality, Total mortality, Cohort

## Abstract

**Background:**

Traffic air pollution has been linked to cardiovascular mortality, which might be due to co-exposure to road traffic noise. Further, personal and lifestyle characteristics might modify any association.

**Methods:**

We followed up 52 061 participants in a Danish cohort for mortality in the nationwide Register of Causes of Death, from enrollment in 1993–1997 through 2009, and traced their residential addresses from 1971 onwards in the Central Population Registry. We used dispersion-modelled concentration of nitrogen dioxide (NO_2_) since 1971 as indicator of traffic air pollution and used Cox regression models to estimate mortality rate ratios (MRRs) with adjustment for potential confounders.

**Results:**

Mean levels of NO_2_ at the residence since 1971 were significantly associated with mortality from cardiovascular disease (MRR, 1.26; 95% confidence interval [CI], 1.06–1.51, per doubling of NO_2_ concentration) and all causes (MRR, 1.13; 95% CI, 1.04–1.23, per doubling of NO_2_ concentration) after adjustment for potential confounders. For participants who ate < 200 g of fruit and vegetables per day, the MRR was 1.45 (95% CI, 1.13–1.87) for mortality from cardiovascular disease and 1.25 (95% CI, 1.11–1.42) for mortality from all causes.

**Conclusions:**

Traffic air pollution is associated with mortality from cardiovascular diseases and all causes, after adjustment for traffic noise. The association was strongest for people with a low fruit and vegetable intake.

## Background

Although several recent studies have shown associations between long-term exposure to traffic-related air pollution and mortality from cardiovascular disease and all causes [1-9], several questions remain open. Exposure to road traffic noise might explain the observed associations, as this has been associated with morbidity and mortality from cardiovascular disease [10]. Furthermore, air pollution could affect the risk for cardiovascular disease through mechanisms involving systemic oxidative stress and inflammation, which could drive atherosclerosis progression and other long-term effects as well as serve as triggers of events through changes in vascular function, thrombogenecity, plaque stability and autonomic balance [11]; the amount of fruit and vegetables in the diet, containing antioxidants and related compounds, might therefore modify the effect of air pollution as suggested for short-term mortality in a case-crossover study in Hongkong [12]. People with pre-existing cardiovascular disease or diabetes mellitus might be particularly susceptible to the effects of air pollution on cardiovascular mortality. Exposure to air pollution decades back in time and perhaps throughout life might be important in the development of chronic cardiovascular disease [13]. Most previous studies of long-term exposure, however, have focused on the addresses of participants at baseline, and few studies have investigated exposure assessed from address history [4,6,14,15].

We report here the results of a Danish cohort study of the a-priori hypothesis that mortality from cardiovascular disease and all causes is associated with long-term exposure to traffic-related air pollution at the residence, derived from residential histories from 1971 onwards. Road traffic noise and other potential confounders were adjusted for, and possible effect modification by personal and lifestyle characteristics was investigated.

## Methods

Design and study participants. Between 1993 and 1997, a population-based sample of 57 053 men (48%) and women (52%) aged 50–64 years and living in the Copenhagen and Aarhus areas, born in Denmark and with no previous cancer diagnosis, were enrolled into the Diet, Cancer and Health cohort study [16]. The examination at baseline, i.e. enrollment, included a self-administered questionnaire on average dietary habits over the last year, which covered 192 food and beverage items. The participants also filled in a questionnaire on smoking habits (status, intensity and duration), occupation, length of school attendance, physical activity, history of diseases and medication, and a number of other health-related items [16]. Staff in the study clinics obtained anthropometric measurements, including height and weight. The average gross income in the municipality of residence at the time of enrollment was provided by Statistics Denmark. Relevant Danish ethical committees and data protection agencies approved the study, and written informed consent was obtained from all participants.

Each cohort member was followed up for death, including date and underlying cause, from cardiovascular disease (ICD-10 codes I00–I99), from the date of inclusion into the cohort until 31 December 2009 in the Danish Register of Causes of Death, by use of the unique personal identification number [17]. Participants who died of external causes (ICD-10 codes S–Z) were censored at the date of death. We extracted the date of emigration or disappearance and the addresses of all cohort members between 1 January 1971 and 31 December 2009 from the Central Population Registry by use of the personal identification number, including the dates of moving to and from each address. The addresses were linked to the Danish address database to obtain geographical coordinates (‘geocodes’), which were obtained for 94% of the addresses.

Exposure assessment. The outdoor concentration of nitrogen dioxide (NO_2_) was calculated at the residential addresses of each cohort member with the Danish AirGIS dispersion modeling system (see http://www.dmu.dk/en/air/models/airgis/). AirGIS is based on a geographical information system (GIS) and provides estimates of traffic-related air pollution with high temporal and address-level spatial resolution. Air pollution at a location was calculated as the sum of: (1) local air pollution from street traffic, calculated from traffic (intensity and type), emission factors for the car fleet, street and building geometry and meteorology; (2) urban background, calculated from data on urban vehicle emission density, city dimensions and building heights; and (3) regional background, estimated from trends at rural monitoring stations and from national vehicle emissions. With the geocode of an address and a specified year as the starting point, the AirGIS system automatically generates street configuration data for the street pollution model, including street orientation, street width, building heights in wind sectors, amount of traffic, speed and type as well as other required data.

The AirGIS system has been validated in several studies [18-21], and the correlation between modelled and measured half-year mean NO_2_ concentrations at 204 positions in the greater Copenhagen area showed a correlation coefficient of 0.90, measured concentrations being on average 11% lower than those modelled [20]. We also compared modelled and measured 1-month mean concentrations of NO_x_ and NO_2_ over 12 years (1995–2006) on a busy street in Copenhagen (Jagtvej, 25 000 vehicles per day, street canyon), with correlation coefficients of 0.88 for NO_x_ and 0.67 for NO_2_. The modelled mean concentration over the whole 12-year period was 6% lower than the measured concentrations of NO_x_ and 12% lower than those of NO_2_[21]. Thus, the model predicted both geographical and temporal variation well.

We used the concentration of NO_2_ as an indicator of air pollution from traffic. We calculated the yearly averages of NO_2_ concentration at all addresses from 1 January 1971 until date of death, censoring or end of follow-up and entered time-weighted average NO_2_ concentration from 1971 as a time-dependent variable into the statistical risk model, thus recalculating exposure for survivors at the time of each death. If an address could not be geocoded, the preceding address was used for NO_2_ calculation; if the first address was missing, the subsequent address was used. We included only participants for whom the residential addresses were known and geocoded for 80% or more of the time from 1 January 1971 to death, censoring or end of follow-up.

Potential confounders and effect modifiers. We defined potential confounding factors a priori from evidence of an association with mortality and modeled them as categorical or continuous. The continuous variables were modeled as linear or a non-linear cubic spline function. The covariates, assessed at baseline, were: sex; calendar year (spline); unemployment during year before enrollment (yes/no); length of school attendance (< 8, 8–10 and > 10 years); risky occupation, defined as job held for a minimum of 1 year with potential exposure to smoke, particles, fumes or chemicals (yes/no) (mining, rubber industry, tannery, chemical industry, wood-processing industry, metal processing [welding, painting, electroplating], foundry, steel-rolling mill, shipyard, glass industry, graphics industry, building industry [roofer, asphalt worker, demolition worker], truck, bus or taxi driver, manufacture of asbestos or asbestos cement, asbestos insulation, cement article industry, china and pottery industry, painter, welder, hairdresser, auto mechanic); smoking status (never, former, current); smoking intensity (lifetime average, spline, calculated by equating a cigarette to 1 g, a cheroot or a pipe to 3 g, and a cigar to 4.5 g of tobacco); smoking duration (total number of years smoking, linear) (smoking status, intensity and duration were adjusted for as three separate variables); environmental tobacco smoke (indicator of exposure, e.g. “smoker in the home or/and exposure at work for at least 4 h/day”); physically active sport (categorical yes/no indicator and linear intensity among active people); body mass index (spline); waist circumference (linear); alcohol intake (categorical yes/no indicator and spline for intensity among drinkers); fat intake (linear); fruit and vegetable intake (linear); fiber intake (linear); fish intake (linear); folate intake (linear); use of hormone replacement therapy (categorical yes/no indicator and linear duration among users); noise at the baseline address (linear); and average gross income in 1995 in the municipality of residence at the time of enrollment (spline).

Road traffic noise was calculated as the A-weighted sound pressure level at the most exposed facade of the baseline residence during the day, evening and night, expressed as L_den_ as an indicator of the overall noise level during 24 h, with a 5 dB penalty for the evening and a 10 dB penalty for the night [22]. We used the noise calculation software Soundplan (version 6.5, http://www.soundplan.dk) and the joint Nordic prediction method for road traffic noise, which has been the standard method for noise calculation in Scandinavia for many years; see details elsewhere [22]. The prespecified potential effect modifiers were: sex, educational level, body mass index, physical activity, intake of fruit and vegetables, smoking status and pre-existing morbidity at baseline.

**Figure 1:**
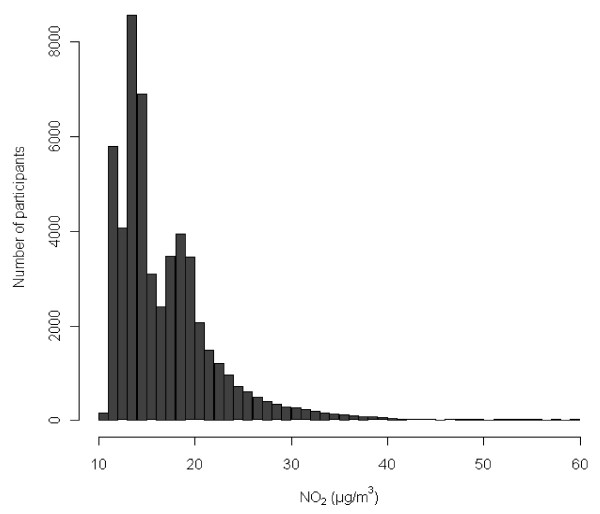
**Distribution of NO**_**2**_**.** Time-weighted average concentrations of NO_2_ at the residential addresses of 52 061 cohort participants from 1971 onwards.

Statistical methods. Mortality rate ratios (MRRs) were estimated from Cox proportional hazards models with Stata 11.0 and left truncation, with age as the time scale. Participants were censored at the time of loss to follow-up due to emigration or disappearance or 31 December 2009, whichever came first. NO_2_ was modeled as a time-dependent variable. The distribution of NO_2_ levels at addresses from 1971 until death or censoring was right-skewed (Figure 1); we log-transformed the NO_2_ concentration using logbase 2, corresponding to interpretation of MRRs as “per doubling of exposure” to avoid excessive influence from observations in the right tail of the distribution and because the NO_2_-mortality function fitted better to a linear model after log-transformation of NO_2_. We investigated the shape of the exposure–mortality function for each continuous potential confounder using cubic splines to determine whether the variable should be modeled as linear or as a spline in the final models.

We investigated interactions with the likelihood ratio test, comparing models with and without an interaction term. The potential effect modifiers were tested one at a time in the fully adjusted model. Marital status (single, married, divorced, widow or widower) did not fulfill the proportional hazard assumption and, therefore, we did not adjust for this variable. Instead we specified separate baseline hazards for each level of marital status (stratified Cox model). Exposure–response functions with 95% confidence limits (CIs) were estimated and visualized using restricted cubic splines (library Survival and library Design in R statistical software 2.9.0) adjusting for the potential confounders.

We used 5% as level of significance.

Sensitivity analyses. We tested the sensitivity to alternative exposure definitions, adjustment for pre-existing disease, use of non-logged NO_2_ concentrations and use of frailty models with municipality as a random effect to take into account spatial correlation at municipality level (see Additional file 1: Supplemental methods).

## Results

Of 57 053 enrolled cohort members, 571 were excluded because of a cancer diagnosis before baseline, two because of uncertain date of cancer diagnosis, 960 for whom an address history was not available in the Central Population Registry or their address at baseline could not be geocoded, 948 because exposure was assessed for less than 80% of the time between 1 January 1971 and death or censoring, and 2511 for whom a value was missing for a potential confounder or effect modifier, leaving 52 061 cohort members for the study. These participants were followed up for an average of 13.0 years, during which time 5534 died from non-external causes, providing a crude mortality rate of 817 per 100 000 person–years at risk.

Table 1 and Table S1 (see Additional file 2: Table S1) show the baseline characteristics of the 52 061 cohort members, who were on average 56.7 years old, with slightly more women than men. Compared to the whole cohort, those who died during follow up had shorter school attendance, more were divorced, unemployed, occupationally exposed to air pollution, smokers and exposed to environmental tobacco smoke, had a higher intake of fat, a lower intake of fruit and vegetables, were less physically active, had pre-existing cardiovascular disease and were living close to dense traffic and in a municipality with low average income. Furthermore, among those living at locations with high NO_2_ levels, more were single or divorced, were smokers and exposed to environmental tobacco smoke, less physical activity, used hormone replacement therapy and were exposed to a higher noise level; many characteristics were, however, similar for people living at residences with high and low levels of NO_2_. The mean NO_2_ concentration at the residences of all participants after 1971 was 16.9 μg/m^3^ (minimum, 10.5 μg/m^3^; median, 15.1 μg/m^3^; maximum, 59.6 μg/m^3^), with similar mean and median values for participants living in municipalities below the median income level (16.8 and 14.5 μg/m^3^) and above the median income level (16.9 and 15.9 μg/m^3^). Noise at the baseline addresses of the study participants correlated with the NO_2_ measures: Spearman’s correlation coefficient (*r*_*s*_) = 0.59 in comparison with the average NO_2_ at all addresses after 1971 and *r*_*s*_ = 0.64 in comparison with NO_2_ at the baseline address.

**Table 1:** **Characteristics of 52 061 study participants, those who died during follow-up and those exposed to low and high levels of NO **_**2 **_**at their residences (See Additional file **2**: Table S1, Additional file **2** for further characteristics)**

**Characteristic**^**a**^	**Cohort**		**All-cause deaths**^**b**^		**NO**_**2**_^**c**^**<19.0 μg/m**^**3**^		**NO**_**2**_**≥19.0 μg/m**^**3**^	
	**% (No.)**	**Median (5–95 percentile)**	**% (No.)**	**Median (5–95 percentile)**	**% (No.)**	**Median (5–95 percentile)**	**% (No.)**	**Median (5–95 percentile)**
All participants	100% (52 061)		10.6% (5534)		75.0% (39 045)		25.0% (13 016)	
Age at baseline (years)		56.1 (50.7-64.1)		59.1 (51.2-64.7)		56.1 (50.7-64.1)		56.2 (50.7-64.2)
Sex								
Male	47.5% (24 734)		59.5% (3 292)		48.0% (18 734)		46.1% (6 000)	
Female	52.5% (27 327)		40.5% (2 242)		52.0% (20 311)		53.9% (7 016)	
School attendance (years)								
< 8	32.8% (17 064)		42.2% (2 349)		32.4% (12 653)		33.9% (4 411)	
8-10	46.2% (24 066)		41.1 % (2 274)		46.5% (18 169)		45.3% (5 897)	
> 10	21.0% (10 931)		16.5% (911)		21.1% (8 223)		20.8% (2 708)	
Body mass index (kg/m^2^)		25.5 (20.4-33.3)		26.0 (19.8-34.6)		25.5 (20.5-33.1)		25.6 (20.3-33.9)
Physical activity (sport)								
No	45.7% (23 787)		60.4% (3 345)		43.8% (17 104)		51.3% (6 683)	
Yes (h/week)	54.3% (28 274)	2.0 (0.5-7.0)	39.6% (2 189)	2.0 (0.5-7.0)	56.2% (21 941)	2.0 (0.5-6.5)	48.7% (6 333)	2.0 (0.5-7.0)
Smoking								
Never	36.0% (18 766)		18.4% (1 021)		37.5% (14 667)		31.5% (4 099)	
Former	27.6% (14 354)		22.6% (1 249)		28.5% (11 107)		24.9% (3 247)	
Current	36.4% (18 941)		59.0% (3 264)		34.0% (13 271)		43.5% (5 670)	
Intensity (g/day) ^d^		14.8 (3.8-34.4)		17.3 (6.0-36.7)		14.6 (3.7-34.6)		15.2 (4.0-34.1)
Duration (years) ^d^		33.0 (7.0-46.0)		38.0 (12.0-49.0)		32.0 (7.0-46.0)		34.0 (8.0-46.0)
Fruit and vegetable intake (g/day)		312 (96.0-734)		265 (71.8 -704)		315 (101-726)		301 (85.0 -754)
Cardiovascular disease at enrolment (any of the five below)	23.1% (12 015)		33.0% (1 828)		23.0% (8 973)		23.4% (3 042)	
Myocardial infarction	2.0% (1 061)		6.4% (356)		2.0% (768)		2.2% (293)	
Angina pectoris	3.1% (1 604)		6.3% (348)		3.0% (1 190)		3.2% (414)	
Stroke	1.3% (682)		3.4% (187)		1.3% (498)		1.4% (184)	
Hypertension	16.3% (8 485)		22.6% (1 251)		16.1% (6 303)		16.8% (2 182)	
Hypercholesterolemia	7.4% (3 880)		10.0% (554)		7.6% (2 985)		6.9% (895)	
Diabetes mellitus at baseline	2.0% (1 069)		5.1% (284)		1.9% (754)		2.4% (315)	
NO_2_ at front door (μg/m^3^) since 1971^c^		15.1 (11.5-27.1)		16.6 (11.6-29.5)		14.2 (11.4-18.5)		22.1 (19.2-34.8)
Major road^e^ within 50 m of address at baseline								
No	92.0% (47 886)		89.6% (4 958)		97.0% (37 856)		77.1% (10 030)	
Yes	8.0% (4 175)		10.4% (576)		3.0% (1 189)		22.9% (2 986)	
Traffic load within 200 m of the address at baseline (10^3^ vehicle km/day)		2.5 (0.3-15.1)		3.5 (0.3-16.1)		1.7 (0.2-12.0)		6.9 (0.6-22.9)
Noise (L_*den*_) at baseline address (dB)		56.4 (48.4-70.0)		57.9 (48.9-71.0)		54.6 (48.0-66.3)		63.4 (52.2-73.1)

^a^ At baseline unless otherwise specified.

^b^Excluding external cause of death.

^c^Time-weighted average for the period 1 January 1971 to death, censoring or end of follow-up.

^d^Based on all people who had ever smoked; lifetime average smoking intensity.

^e^More than 10 000 vehicles per day.

**Figure 2:**
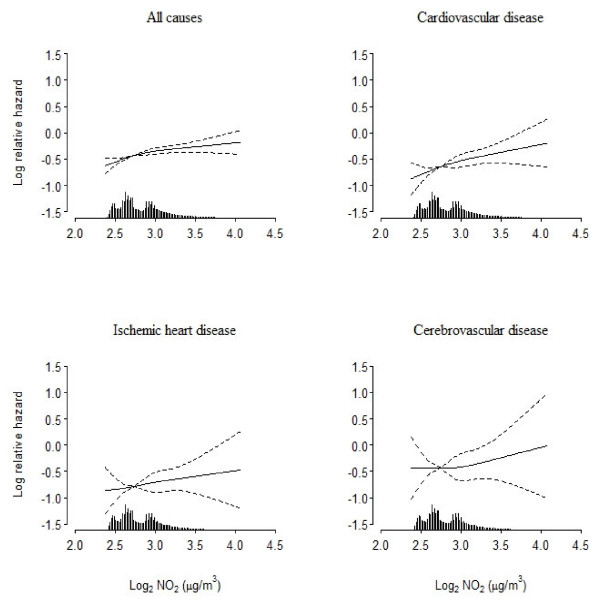
**Spline functions between NO**_**2 **_**and mortality.** Spline functions (filled lines; 95% confidence limits indicated by dashed lines) between average NO_2_ concentration (μg/m^3^) at residences from 1971 onwards and mortality from all causes and cardiovascular disease. Functions adjusted for the same potential confounders as those relevant for results in the last column of Table 2.

Table 2 shows that NO_2_ at the residence after 1971 was associated with mortality from cardiovascular disease and all causes. The MRRs for the different causes of death ranged from 1.40 to 2.50 in association with a doubling of the NO_2_ concentration in the basic model, with adjustment for age and sex. All MRRs were attenuated by further adjustment for various covariates; additional adjustment for road traffic noise at the enrollment address further attenuated the MRRs, although only marginally for mortality from cerebrovascular disease and ‘other’ cardiovascular diseases. In the fully adjusted model, a doubling of the NO_2_ concentration at the residence was associated with a 26% (95% CI, 6–51%) higher cardiovascular mortality rate, a 71% (95% CI, 25-137%) higher ‘other’ cardiovascular mortality rate and a 13% (95% CI, 4–23%) higher all-cause mortality rate. Figure 2 shows almost linear exposure–response functions between log-NO_2_ and MRRs for all cardiovascular disease, ischemic heart disease and all causes. Tentative adjustment for pre-existing morbidity at baseline provided virtually identical results (results not shown).

**Table 2:** **Mortality rate ratios associated with time-weighted average concentration of NO**_**2 **_**from 1971 onwards at residential addresses**

		**Mortality rate ratio**^**a**^**(95% CI)**		
**Mortality (ICD-10 codes)**	**N**_**deaths**_	**Model with adjustment for sex and age**^**b**^	**Model with further adjustment for various variables**^**c**^	**Model with further adjustment for noise**^**d**^
All causes (except external, S-Z)	5534	1.52 (1.42-1.62)	1.18 (1.10-1.26)	1.13 (1.04-1.23)
Cardiovascular (I00-99)	1285	1.71 (1.50-1.94)	1.33 (1.16-1.54)	1.26 (1.06-1.51)
Ischemic heart disease (I20-25)	548	1.48 (1.21-1.82)	1.23 (0.99-1.54)	1.12 (0.85-1.47)
Cardiac rhythm disturbances (I44 + I47-49)	25	2.32 (0.95-5.67)	1.41 (0.50-3.94)	1.01 (0.28-3.65)
Heart failure (I50)	44	1.89 (0.94-3.80)	1.14 (0.52-2.51)	0.94 (0.35-2.53)
Cerebrovascular disease (I60-69)	292	1.40 (1.06-1.86)	1.13 (0.83-1.53)	1.11 (0.78-1.63)
Other cardiovascular disease	376	2.46 (1.96-2.09)	1.80 (1.41-2.32)	1.71 (1.25-2.37)

Results based on 677 761 person-years at risk for 52 061 cohort participants from baseline (1993-1997) through 2009.

^a^Given per doubling of the NO_2_ concentration.

^b^Adjusted for sex and age (age was the time scale in the Cox models).

^c^ Adjusted for sex, age (age was the time scale), calendar year, employment status, school attendance, occupation with potential exposure to smoke and fumes, smoking status, smoking intensity, smoking duration, environmental tobacco smoke, alcohol, fat, fish, fruit and vegetables, fiber, folate, body mass index, waist circumference, physical activity with sport, hormone replacement therapy, average gross income of municipality of residence in 1995. The Cox model stratified for marital status.

^d^As previous model with further adjustment for noise at the baseline address.

We compared the results based on our primary exposure measure (NO_2_ since 1971) with those for four alternative exposure measures: NO_2_ since 1991, NO_2_ at the baseline address, presence of a major road within 50 m and total traffic load within 200 m of the baseline address (Table 3). The two long-term NO_2_ measures (NO_2_ since 1971 and 1991) showed the strongest associations with mortality; NO_2_ at the baseline address showed weaker associations, and the two measures of traffic at the baseline address showed even weaker associations. The results for the subcohort living at the baseline address throughout the followup period were virtually identical ( Additional file 3: Table S2).

**Table 3:** Mortality rate ratios associated with different exposure measures at residential addresses

	**Mortality rate ratio**^**a **^**(95% confidence interval)**				
**Exposure**	**All causes (n = 5534)**	**Cardiovascular disease (n = 1285)**	**Ischemic heart disease (n = 548)**	**Cerebrovascular disease (n = 292)**	**Other cardiovascular disease (n = 376)**
NO_2_ from 1971 onwards^b^	1.13 (1.04-1.23)	1.26 (1.06-1.51)	1.12 (0.85-1.47)	1.11 (0.76-1.63)	1.72 (1.25-2.37)
NO_2_ from 1991 onwards^b^	1.13 (1.05-1.22)	1.21 (1.02-1.42)	1.13 (0.88-1.45)	0.99 (0.70-1.41)	1.56 (1.17-2.10)
NO_2_ (1-year mean) at address at baseline^b^	1.09 (1.01-1.19)	1.16 (0.99-1.37)	1.09 (0.85-1.41)	1.06 (0.75-1.52)	1.42 (1.06-1.92)
Major road within 50 of address at baseline	0.94 (0.85-1.05)	0.98 (0.79-1.21)	1.04 (0.76-1.44)	0.87 (0.54-1.39)	1.03 (0.71-1.49)
Traffic load within 200 m of address at baseline^c^	1.01 (0.99-1.03)	1.02 (0.98-1.06)	1.01 (0.95-1.07)	1.02 (0.94-1.11)	1.03 (0.96-1.11)

Results based on 677 761 person–years at risk for 52 061 cohort participants from baseline (1993–1997) through 2009.

^a^Adjusted for sex, age (age was the time scale), calendar year, employment status, school attendance, occupation with potential exposure to smoke and fumes, smoking status, smoking intensity, smoking duration, environmental tobacco smoke, alcohol, fat, fish, fruit and vegetables, fiber, folate, body mass index, waist circumference, physical activity with sport, hormone replacement therapy, average gross income of municipality of residence in 1995 and noise at the baseline address. The Cox model stratified for marital status.

^b^The mortality rate ratio is given per doubling of the NO_2_ concentration. The three NO_2_ measures correlated strongly; r_s_ = 0.92 between NO_2_ from1971 and NO_2_ from 1991; r_s_ = 0.87 between NO_2_ from1971 and NO_2_ at baseline; r_s_ = 0.95 between NO_2_ from1991 and NO_2_ at baseline.

^c^The mortality rate ratio is given per doubling of the traffic load.

**Figure 3:**
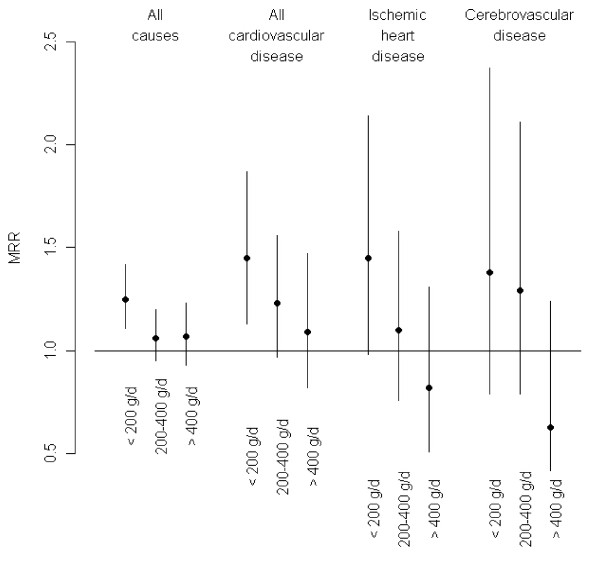
**Mortality rate ratios by intake of fruit and vegetables.** Mortality rate ratios (MRR, dots) with 95% confidence intervals (whiskers) for all causes, all cardiovascular disease, ischemic heart disease and cerebrovascular disease associated with NO_2_ concentrations at residences since 1971, by three levels of intake of fruit and vegetables.

Figure 3 and Table 4 show effect modification by intake of fruit and vegetables, which was consistent for all three cardiovascular mortality end-points: the MRRs were highest for people with low intake of fruit and vegetables (< 200 g/day), intermediate for those eating 200–400 g fruit and vegetables per day and lowest for those with a high intake (> 400 g/day). The results showed no clear differences in MRRs between people with and without pre-existing morbidity at baseline or any of the other potential effect modifiers (Table 4). Additional file 4: Table S3, gives the numbers of deaths and person–years at risk corresponding to the cells in Table 4.

**Table 4:** **Mortality rate ratios associated with NO**_**2 **_**at the front door from 1971 onwards among 52 061 cohort participants, by potential effect modifiers**

**Potential effect modifier**	**Covariate level**	**Mortality rate ratio (95% CI)**^**a**^			
		**All causes**	**Cardiovascular disease**	**Ischemic heart disease**	**Cerebrovascular disease**
Whole cohort^b^		1.13 (1.04-1.23)	1.26 (1.06-1.51)	1.12 (0.85-1.47)	1.11 (0.78-1.63)
Sex	Male	1.19 (1.07-1.32)	1.28 (1.05-1.56)	1.15 (0.85-1.54)	1.31 (0.84-2.04)
	Female	1.05 (0.94-1.19)	1.22 (0.93-1.60)	1.03 (0.65-1.53)	0.89 (0.53-1.50)
	p for interaction	0.08	0.74	0.66	0.20
School attendance (years)	< 8	1.15 (1.03-1.29)	1.25 (1.00-1.56)	1.07 (0.76-1.50)	1.13 (0.68-1.87)
	8-10	1.16 (1.03-1.30)	1.34 (1.05-1.71)	1.17 (0.80-1.71)	1.02 (0.61-1.71)
	> 10	0.99 (0.83-1.19)	1.11 (0.76-1.61)	1.20 (0.65-2.23)	1.34 (0.64-2.78)
	p for interaction	0.22	0.79	0.65	0.80
Body mass index (kg/m^2^)	< 25	1.12 (1.00-1.26)	1.13 (0.90-1.55)	1.14 (0.74-1.74)	0.99 (0.58-1.68)
	25-30	1.15 (1.02-1.29)	1.33 (1.06-1.67)	1.22 (0.85-1.73)	1.31 (0.81-2.13)
	> 30	1.13 (0.96-1.32)	1.24 (0.93-1.66)	0.96 (0.62-1.49)	0.92 (0.42-1.99)
	p for interaction	0.91	0.84	0.51	0.90
Physical activity (sport)	No	1.17 (1.05-1.29)	1.25 (1.02-1.53)	1.15 (0.84-1.56)	1.05 (0.68-1.64)
	Yes	1.08 (0.95-1.22)	1.29 (1.01-1.66)	1.07 (0.72-1.59)	1.22 (0.72-2.07)
	p for interaction	0.25	0.80	0.75	0.63
Fruit and vegetable consumption (g/day)	< 200	1.25 (1.11-1.42)	1.45 (1.13-1.87)	1.45 (0.98-2.14)	1.38 (0.79-2.37)
	200-400	1.06 (0.95-1.20)	1.23 (0.97-1.56)	1.10 (0.76-1.58)	1.29 (0.79-2.11)
	> 400	1.07 (0.93-1.23)	1.09 (0.82-1.47)	0.82 (0.51-1.31)	0.63 (0.32-1.24)
	p for interaction	0.04	0.12	0.05	0.08
Smoking status	Never	1.18 (1.00-1.39)	1.29 (0.90-1.85)	1.35 (0.78-2.35)	0.79 (0.37-1.70)
	Former	1.05 (0.90-1.22)	1.02 (0.75-1.39)	0.97 (0.62-1.53)	0.99 (0.50-1.96)
	Current	1.15 (1.04-1.27)	1.36 (1.11-1.67)	1.13 (0.82-1.56)	1.27 (0.82-1.97)
	p for interaction	0.94	0.39	0.78	0.22
Pre-existing morbidity^c^ at baseline	No	1.13 (0.99-1.28)	1.43 (1.15-1.79)	1.38 (0.97-1.96)	1.18 (0.74-1.86)
	Yes	1.15 (1.04-1.26)	1.17 (0.94-1.60)	1.00 (0.72-1.40)	1.09 (0.66-1.79)
	p for interaction	0.79	0.13	0.13	0.79

^a^Per doubling of NO_2_ concentration. Adjusted for sex, age (age was the time scale), calendar year, employment status, school attendance, occupation with potential exposure to smoke and fumes, smoking status, smoking intensity, smoking duration, environmental tobacco smoke, alcohol intake, fat, fish, fruit and vegetables, fiber, folate, body mass index, waist circumference, physical activity with sport, hormone replacement therapy, average gross income of municipality of residence in 1995 and noise at the baseline address. The Cox model stratified for marital status.

^b^Identical to estimates in the last column of Table 2; shown here for comparison.

^c^Myocardial infarction, angina pectoris, stroke, hypertension, hypercholesterolemia or diabetes mellitus. The model included adjustment for main effects of pre-existing morbidity.

NO_2_ concentration at the residences since 1971, without log-transformation, was associated with a 16% (95% CI, 3–31%) higher cardiovascular mortality rate and an 8% (95% CI, 1–14%) higher all-cause mortality rate per 10 μg/m^3^ NO_2_ ( Additional file 5:Table S4).

Frailty models with municipality included as a random effect indicated area level confounding for all cause but not for cardiovascular mortality ( Additional file 6: Table S5).

## Discussion

We found associations between long-term measures of traffic-related air pollution at the residence and mortality from cardiovascular disease and all causes, in agreement with previous studies [1-9]. Adjustment for road traffic noise attenuated the estimated MRRs, but associations with NO_2_ concentration remained. The association between NO_2_ and mortality was strongest for people with the lowest intake of fruit and vegetables and weakest (or absent) for people with the highest intake.

The strengths of this study include a 13-year prospective follow-up of a large cohort and adjustment for road traffic noise and other potential confounders. Follow-up for cause-specific mortality and vital status was possible through nationwide population-based registries. Further, exposure assessment at individual addresses allowed detection of within-city contrasts, which might be more strongly associated with cardiovascular events than between-city contrasts [23,24]. The model used to calculate NO_2_ concentrations at addresses requires comprehensive input data and has been validated [19-21] and applied [25-27]. Although model-based estimates of air pollution concentrations are inevitably somewhat uncertain, any resulting non-differential misclassification would create artificial associations only in rare situations [28]. The data on mortality were from the Danish Registry of Causes of Death, and the underlying cause of death was defined from information on death certificates [17]. A validation study showed that the Danish Registry of Causes of Death has a predictive value of 70% and a sensitivity of 81% for death due to acute myocardial infarction [29]. Misclassification of the underlying cause of death is unlikely to be associated with air pollution levels and would change the MRRs towards 1.00 rather than create artificial associations. Personal characteristics of the participants were collected at baseline. Some factors (e.g. smoking duration and intensity, educational level and HRT use) covered the whole life until baseline; others covered a shorter period (e.g. dietary habits which covered the last year before baseline); and others (such as BMI and waist circumference) referred to one point in time (baseline). It is uncertain to which degree the collected information covers also the time after baseline and for e.g. diet and BMI also the time many years before baseline. The study population was between 50 and 64 years old at baseline, and lifestyle at these ages are usually relatively stable and representative for the decades before and after. However, participants who developed a disease after baseline might indeed have changed lifestyle, which might cause misclassification when using baseline characteristics.

Previous studies of NO_2_ and mortality from cardiovascular disease and all causes have shown both stronger [3,8,14,30], similar [4,8,31,32] and weaker [2,8,9] associations than this study when comparing effect estimates corresponding to 10 μg/m^3^ NO_2_. The differences might be due to different methods or differences in the air pollution mixture for which NO_2_ is a marker. The confidence intervals of the present study overlap widely with those of corresponding results from the previous studies indicating that chance might also explain the differences.

Several risk factors for mortality, such as length of school attendance, smoking and physical activity, were associated with NO_2_ levels at the residence, and adjustment for these (and other) factors reduced the MRRs substantially, as expected. Exposure to road traffic noise is associated with both traffic-related air pollution and cardiovascular health [10] and was therefore also a potential confounder in the present study. Although adjustment for road traffic noise reduced the risk estimates associated with NO_2_, the effects on mortality from cardiovascular disease and all causes remained. An effect of air pollution on mortality from cardiovascular disease independent of concomitant noise is in line with the results of two recent studies [33,34].

It is uncertain which period of exposure is relevant for an association between exposure to air pollution and morbidity and mortality from cardiovascular disease. We found stronger associations of mortality from all cardiovascular disease with exposure since 1971 and 1991 than with 1-year average exposure at the baseline address, although the difference was small. This might indicate the relevance of decades of exposure, perhaps explained by effects of air pollution on the chronic process of atherogenesis [13] or other mechanisms of importance for the development of cardiovascular diseases [11]. Our study addressed long-term exposure; however, people living in highly polluted areas are probably also more likely to be exposed to high peak exposures. Strong correlations (*r*_*s*_ between 0.87 and 0.95) for NO_2_ over the three periods precluded a more detailed analysis of the effect of timing of exposure in the present study.

In contrast to our findings with modeled NO_2_ at residences, we found no significant associations with indicators of traffic at the residence. This difference might be due to the fact that the air pollution model takes into account a number of factors of relevance for the air pollution concentration (such as street width, building geometry, amount, type, speed and emission factors of traffic, background contributions), providing a more precise assessment of air pollution than the simple traffic counts used for the traffic indicators. Previous studies have shown associations with simple traffic indicators, however without adjustment for road traffic noise [2,6,35,36]. Post-hoc analyses without adjustment for noise showed associations between the simple traffic indicators and mortality from cardiovascular disease and all causes ( Additional file 7: Table S6). Thus, when NO_2_ and noise were assessed in state-of-the art exposure models with extensive input data of similar quality, significant associations were found between NO_2_ concentration and mortality from cardiovascular disease and all causes also after adjustment for road traffic noise. When the simple, less precise proxy measures of air pollution, traffic indicators, were adjusted for the more precisely determined street noise levels, the estimated effect of traffic might be ‘over-adjusted’.

We adjusted for noise at the baseline address even when estimating effects of air pollution over much longer time periods, because noise calculations were not available at all addresses since 1971. This might imply insufficient adjustment for noise, i.e. residual confounding. However, the results also showed a significant effect of air pollution after adjustment for noise when estimating both air pollution and noise at the baseline address and restricting to cohort participants who lived at the same address from baseline onwards ( Additional file 3: Table S2).

Dietary intake of fruit and vegetables modified the association between NO_2_ and mortality, so that the association was strongest for people with a low intake of fruit and vegetables and weakest (or absent) among people with a high intake. This is in line with a case-crossover study of short-term effects of air pollution, which showed the strongest effects on mortality among those with a low intake of fruit and vegetables [12]. We found associations between NO_2_ concentration and mortality; NO_2_ is not only an airway irritant but also an indicator of vehicle engine exhaust, which is a complex mixture of many chemicals, including particulate matter with absorbed polycyclic aromatic hydrocarbons, quinones, transition metals and other substances. Thus, associations observed between NO_2_ and cardiovascular diseases might be caused by multiple of these correlated substances, which in general can cause oxidative stress and inflammation, which in turn can promote cardiovascular disease mechanisms including short-term related endothelial dysfunction, plaque rupture, thrombogenecity and autonomic imbalance and long-term related atherosclerosis progression, plague instability, insulin resistance and dyslipidemia [11,13,37,38]. A possible mechanism for a protective effect of fruit and vegetables that are rich in antioxidants and related compounds is scavenging of free radicals and reactive oxygen species generated by exposure to air pollution before they can affect vascular function, oxidize lipids and activate proinflammatory, prothrombotic and other relevant pathways as well as up-regulation of protective enzymes [39-42]. Although a single previous study supports this hypothesis [2], we cannot exclude the possibility that the interaction between intake of fruit and vegetables and mortality from cardiovascular disease observed in this study is a chance finding. Also, a high intake of fruit and vegetables might be an indicator of a generally healthy lifestyle, and the apparent effect modification by fruit and vegetables might be due to other characteristics that were not sufficiently adjusted for in our study. However, the ‘dose–response’ association for three levels of fruit and vegetable intake, the consistency by end-point and biological plausibility speak in favor of a true interaction.

We did not find stronger associations between air pollution and mortality among cohort members with a previous diagnosis of myocardial infarction, angina pectoris, stroke, hypertension, hypercholesterolemia or diabetes mellitus, in line with previous results [8,9,24]. This result, with the finding that adjustment for pre-existing morbidity had virtually no effect on MRRs, indicates that death due to air pollution does not affect only susceptible people with pre-existing cardiovascular disease or diabetes mellitus and that the underlying biological mechanisms of long-term air pollution exposure are general and affect large populations. These conclusions are in line with recent proposals that air pollution promotes the life-long process of atherogenesis and that underlying subclinical atherosclerosis increases the pool of people prone to ‘events’ [13,43].

A previous study indicated a stronger association between air pollution and mortality among women than among men [32], but the results of our and other studies show no such sex difference [4,8,9,44]. Some studies indicated stronger associations between air pollution and cardiovascular events among people with a high body mass index [24,45], which was not confirmed in the present or another study [8]. Two studies suggested that air pollution had the strongest effects on all-cause mortality among people with the lowest educational level [2,44], but our and other studies did not confirm this for all causes [30] or for cardiovascular events [4,24]. Some [2,44,45] but not other studies [8,24,30,46] showed stronger effects of air pollution among people who had never smoked; however, we found no effect modification by smoking status.

Our results show associations between NO_2_ concentration and mortality from ‘other’ cardiovascular diseases, covering a heterogeneous variety of relatively rare causes of death. In view of the large number of other causes of death, the few deaths from each cause and the lack of an a priori hypothesis, we abstained from an explorative analysis for this subgroup.

## Conclusions

In conclusion, this cohort study shows associations between traffic-related air pollution at residential addresses over several decades and mortality from cardiovascular disease and all causes, after adjustment for road traffic noise and other potential confounders. The association between air pollution and mortality was strongest for people with a low intake of fruit and vegetables, which needs confirmation in future studies.

## Abbreviations

CI: Confidence Interval; GIS: Geographical Information System; ICD: International Classification of Diseases; MRR: Mortality Rate Ratio; OSPM: Operational Street Pollution Model; r_s_: Spearman’s Correlation Coefficient.

## Competing interest

The authors have no competing interests.

## Authors’ contributions

ORN conceived and designed the study, participated in acquisition of environmental data and exposure assessment, participated in planning data analyses and drafted the manuscript. ZA participated in planning the statistical analyses, performed record linkages, data processing and statistical analyses. SSJ and MK developed the air pollution modelling system and conducted the air pollution calculations. JH defined the occupations associated with higher mortality. SL contributed to the manuscript. AT and KO established the Diet Cancer and Health cohort and provided cohort data. All authors participated in interpretation of the data, commented on the manuscript and approved the final manuscript for publication.

## Supplementary Material

Additional file 1 Supplemental methods. Click here for file

Additional file 2 **Table S1.** Characteristics of study participants, those who died and those with low and high levels of NO_2_ at their residences. Click here for file

Additional file 3 **Table S2.** Mortality rate ratios associated with different exposure measures at residential addresses, based on cohort participants who lived at the same address from baseline (1993–1997) through 2009. Click here for file

Additional file 4 **Table S3.** Number of deaths and person-years at risk by potential effect modifier among 52 061 participants followed up from baseline (1993–1997). Click here for file

Additional file 5 **Table S4.** Mortality rate ratios in association with non-logged time-weighted average concentration of NO_2_ from 1971 onwards at residential addresses. Click here for file

Additional file 6 **Table S5.** Mortality rate ratios in association with time-weighted average concentration of NO_2_ from 1971 onwards at residential addresses estimated in frailty models with municipality as a random effect. Click here for file

Additional file 7 **Table S6.** Mortality rate ratios associated with exposure measures at residential addresses, based on cohort participants who lived at the same address from baseline (1993–1997) through 2009 and without adjustment for road traffic noise. Click here for file
