# Supplementary material for: Traffic air pollution and mortality from cardiovascular disease and all causes: a Danish cohort study
Source: Environ Health. 2012 Sep 5;11:60. doi: 10.1186/1476-069X-11-60 (PMC3515423; doi:10.1186/1476-069X-11-60)
Supplement: Additional file 1 — Supplemental methods. [file 1476-069X-11-60-S1.pdf]

Supplemental methods

The Danish AirGIS modelling system: input data and application. Input data for the AirGIS system were obtained from various sources and were integrated into the model. A GIS-based road network, including construction year and traffic during the period 1960 onwards, was developed, and a database on emission factors for the Danish car fleet, with data on light- and heavy-duty vehicles back to 1960, was built and entered into the emission module of the street pollution model. The national topographic GIS database of buildings was supplemented by the construction year and building height from the national Building and Dwelling Register, which provided the correct street and building geometry for a given year at a given address. The geocode of an address reflects the location of the front door, usually within 5 m. Air pollution is calculated for 2 m height at the façade of the address building.

The dispersion modelling system has been used in several large epidemiological studies in Denmark [1-6], and the street pollution model, OSPM, which is a core part of AirGIS, has been used in many other countries [7].

The AirGIS modelling system cannot provide reliable estimates of past particulate matter concentrations because the necessary data on previous urban background concentrations and emission factors for the Danish car fleet are not available.

Sensitivity analyses To test the sensitivity for defining exposure, we applied four alternative measures: NO_2 concentration at addresses since 1991, NO_2 at the enrolment address and two simple indicators of traffic at the baseline address: presence of a street with a traffic density > 10 000 vehicles per day within 50 m of the residence, and total number of kilometers driven by vehicles within 200 m of the residence each day. We considered the NO_2 concentration since 1971 as our primary exposure variable, because it takes into account a number of factors that affect traffic-related air pollution and because it reflects exposure over several

decades. We analyzed the five exposure measures for all participants and for a subgroup of participants who had lived at their baseline address throughout the follow-up period.

We did not adjust our main models for pre-existing diseases because we considered that those conditions might be on the causal pathway between exposure to air pollution and death. In a sensitivity analysis, however, we further adjusted for pre-existing myocardial infarction, angina pectoris, stroke, hypertension, hypercholesterolemia or diabetes mellitus at enrolment. Although data fitted better to a linear model after log-transformation of NO_2 levels, we repeated the main analyses for NO_2 without log-transformation and calculated MRRs per 10 $\mu g/m^3$. We also repeated the main analyses fitting frailty models with municipality at baseline as a random effect in addition to the fixed effect of municipality income level in order to take into account spatial correlation at municipality level.

References

1. Raaschou-Nielsen O, Hertel O, Thomsen BL, Olsen JH: **Air pollution from traffic at the residence of children with cancer**. *Am J Epidemiol* 2001, **153**:433-443.

2. Raaschou-Nielsen O, Bak H, Sorensen M, Jensen SS, Ketzel M, Hvidberg M, Schnohr P, Tjonneland A, Overvad K, Loft S: **Air pollution from traffic and risk for lung cancer in three Danish cohorts**. *Cancer Epidemiol Biomarkers Prev* 2010, **19**:1284-1291.

3. Raaschou-Nielsen O, Andersen ZJ, Hvidberg M, Jensen SS, Ketzel M, Sørensen M, Loft S, Overvad K, Tjonneland A: **Lung cancer incidence and long-term exposure to air pollution from traffic**. *Environ Health Perspect* 2011, **119**:860-865.

4. Raaschou-Nielsen O, Andersen ZJ, Hvidberg M, Jensen SS, Ketzel M, Sorensen M, Hansen J, Loft S, Overvad K, Tjonneland A: **Air pollution from traffic and cancer incidence: a Danish cohort study**. *Environ Health* 2011, **10**:67.

5. Andersen ZJ, Hvidberg M, Jensen SS, Ketzel M, Loft S, Sorensen M, Tjonneland A, Overvad K, Raaschou-Nielsen O: **Chronic Obstructive Pulmonary Disease and Long-Term Exposure to Traffic-Related Air Pollution: A Cohort Study**. *Am J Respir Crit Care Med* 2011, **183**:455-461.

6. Andersen ZJ, Bonnelykke K, Hvidberg M, Jensen SS, Ketzel M, Loft S, Sorensen M, Tjonneland A, Overvad K, Raaschou-Nielsen O: **Long-term exposure to air pollution and asthma hospitalisations in older adults: a cohort study**. *Thorax* 2011.

7. Kakosimos KE, Hertel O, Ketzel M, Berkowicz R: **Operational Street Pollution Model (OSPM) - a review of performed application and validation studies, and future prospects**. *Environ Chem* 2010, **7**:485-503.
