# Supplementary material for: Traffic air pollution and mortality from cardiovascular disease and all causes: a Danish cohort study
Source: Environ Health. 2012 Sep 5;11:60. doi: 10.1186/1476-069X-11-60 (PMC3515423; doi:10.1186/1476-069X-11-60)
Supplement: Additional file 2 — Table S1. Characteristics of study participants, those who died and those with low and high levels of NO2 at their residences. [file 1476-069X-11-60-S2.pdf]

Table S1 Characteristics of study participants, those who died and those with low and high levels of NO_2 at their residences

Characteristic[a]	Cohort		Deaths from all causes [b]		NO_2[c] < 19.0 µg/m^3		$NO_2 \geq 19.0$ µg/m^3	
	% (No.)	Median (5–95 percentile)	% (No.)	Median (5–95 percentile)	% (No.)	Median (5–95 percentile)	% (No.)	Median (5–95 percentile)
All participants	100% (52 061)		10.6% (5534)		75.0% (39 045)		25.0% (13 016)	
Marital status								
Single	5.9% (3 067)		6.6% (363)		4.5% (1 743)		10.2% (1 324)	
Married	71.9% (37 454)		65.3% (3 614)		76.1% (29 702)		59.5% (7 752)	
Divorced	16.7% (8 683)		20.7% (1 144)		14.3% (5 567)		23.9% (3 116)	
Widow or widower	5.5% (2 857)		7.5% (413)		5.2% (2 033)		6.3% (824)	
Unemployed last year								
No	78.0% (40 610)		66.0% (3 650)		77.7% (30 334)		78.9% (10 276)	
Yes	22.0% (11 451)		34.0% (1 884)		22.3% (8 711)		21.0% (2 740)	
Risky occupation[d]								
No	66.9% (34 807)		58.2% (3 222)		67.3% (26 268)		65.6% (8 539)	
Yes	33.1% (17 254)		41.8% (2 312)		32.7% (12 777)		34.4% (4 477)	

Environmental tobacco smoke								
No or low	36.0% (18 738)		22.2% (1 230)		37.9% (14 788)		30.3% (3 950)	
High	64.0% (33 323)		77.8% (4 304)		62.1% (24 257)		69.6% (9 066)	
Alcohol intake								
No	2.2% (1 164)		4.3% (241)		2.0% (794)		2.8% (370)	
Yes (g/day)	97.8% (50 897)	13.4 (1.1-64.7)	95.6% (5 293)	15.5 (1.0-88.6)	97.8% (38 251)	13.2 (1.2-62.1)	97.2% (12 646)	13.7 (1.0-75.4)
Fat intake (g/day)		72.4 (39.4-125)		75.5 (40.2-133)		72.5 (39.6-124.1)		71.9 (38.7-127.9)
Fish intake (g/day)		38.1 (11.3-91.7)		39.6 (10.3-99.6)		38.3 (11.6-90.5)		37.7 (10.5-95.6)
Fiber intake (g(day)		20.3 (11.0-33.6)		19.1 (9.6-33.2)		20.5 (11.3-33.6)		19.7 (10.3-34.0)
Folate intake (µg/day)		367 (203-637)		357 (193-647)		367 (204-631)		369 (199-654)
Waist circumference (cm)		89.0 (69.0-116)		92.0 (70.0-116)		89.0 (69.0-110)		88.0 (69.0-111)

Hormone replacement therapy								
Men (never)	47.5% (24 734)		59.5% (3 292)		48.0% (18 734)		46.1% (6 000)	
Women (never)	29.5% (15 338)		21.5% (1 191)		29.9% (11 674)		28.2% (3 664)	
Women (ever, years)	23.0% (11 989)	4.0	19.0% (1 051)	5.0	22.1% (8 637)	4.0	25.7% (3 352)	4.0
		(0.0-18.0)		(0.0-20.0)		(0.0-18.0)		(0.0-19.0)
Municipality income[e]								
< median	48.6% (25294)		55.0% (3046)					
≥ median	51.4% (26767)		45.0% (2488)					

[a] At baseline unless otherwise specified

[b] Excluding external cause of death

[c] Time-weighted average for the period 1 January 1971 to death, censoring or end of follow-up

[d] Ever employed for at least 1 year in an industry or job with potential exposure to dust, smoke or fumes (see Methods for specification)

[e] Average gross income in 1995 of municipality at enrolment; median among participants, 174 100 Dkr
