# Supplementary material for: Traffic air pollution and mortality from cardiovascular disease and all causes: a Danish cohort study
Source: Environ Health. 2012 Sep 5;11:60. doi: 10.1186/1476-069X-11-60 (PMC3515423; doi:10.1186/1476-069X-11-60)
Supplement: Additional file 3 — Table S2. Mortality rate ratios associated with different exposure measures at residential addresses, based on cohort participants who lived at the same address from baseline (1993–1997) through 2009. [file 1476-069X-11-60-S3.pdf]

Table S2. Mortality rate ratios associated with different exposure measures at residential addresses, based on cohort participants who lived at the same address from baseline (1993–1997) through 2009.

Exposure	Mortality rate ratio[a] (95% confidence interval)				
	All causes (n=5182)	Cardiovascular disease (n=1213)	Ischemic heart disease (n=518)	Cerebrovascular disease (n=274)	Other cardiovascular disease (n=357)
NO_2 from 1971 onwards[b]	1.10 (1.01-1.20)	1.23 (1.02-1.48)	1.06 (0.80-1.41)	1.14 (0.77-1.68)	1.67 (1.20-2.32)
NO_2 from 1991 onwards[b]	1.11 (1.02-1.21)	1.22 (1.03-1.44)	1.12 (0.87-1.45)	1.00 (0.69-1.43)	1.62 (1.20-2.20)
NO_2 (1-year mean) at address at baseline[b]	1.09 (1.01-1.19)	1.19 (1.01-1.42)	1.12 (0.86-1.45)	1.09 (0.75-1.57)	1.49 (1.10-2.03)
Major road within 50 m of address at baseline	0.94 (0.85-1.05)	0.95 (0.76-1.18)	1.05 (0.76-1.47)	0.72 (0.43-1.21)	1.05 (0.72-1.52)
Traffic load within 200 m of address at baseline[c]	1.01 (0.99-1.03)	1.02 (0.98-1.07)	1.02 (0.96-1.09)	1.02 (0.94-1.11)	1.04 (0.97-1.13)

Results based on 569 637 person-years at risk for 43 678 cohort participants who lived at the same address from baseline (1993–1997) through 2009

[a] Adjusted for sex, age (age was the time scale), calendar year, employment status, school attendance, occupation with potential for exposure to smoke and fumes, smoking status, smoking intensity, smoking duration, environmental tobacco smoking, alcohol, fat, fish, fruit and vegetables, fiber, folate, body mass index, waist circumference, physical activity with sport, hormone replacement therapy, average gross income of municipality of residence in 1995 and noise at the baseline address. The Cox model stratified for marital status.

[b] The mortality rate ratio is given per doubling of the NO_2 concentration.

[c] The mortality rate ratio is given per doubling of the traffic load
