# Supplementary material for: Traffic air pollution and mortality from cardiovascular disease and all causes: a Danish cohort study
Source: Environ Health. 2012 Sep 5;11:60. doi: 10.1186/1476-069X-11-60 (PMC3515423; doi:10.1186/1476-069X-11-60)
Supplement: Additional file 4 — Table S3. Number of deaths and person-years at risk by potential effect modifier among 52 061 participants followed up from baseline (1993–1997). [file 1476-069X-11-60-S4.pdf]

Table S3 Number of deaths and person-years at risk by potential effect modifier among 52 061 participants followed up from baseline (1993–1997)

Covariate	Covariate level	Person-years at risk	Number of deaths			
			All causes	Cardiovascular disease	Ischemic heart disease	Cerebrovascular disease
Whole cohort		677 761	5534	1285	548	292
Sex	Male	317 226	3292	911	131	168
	Female	360 535	2242	374	417	124
Education (years)	< 8	220 279	2349	584	273	118
	8-10	314 184	2274	503	204	122
	> 10	143 297	911	198	71	52
Body mass index (kg/m^2)	< 25	298 503	2237	397	161	115
	25–30	282 551	2256	566	236	131
	> 30	96 706	1041	322	151	46
Physical activity						

(sport)	No	306 451	3345	799	345	178
	Yes	371 310	2189	486	203	114
Fruit and vegetable intake (g/day)	< 200	157 633	1848	410	170	90
	200-400	296 967	2255	551	240	130
	> 400	223 161	1431	324	138	72
Smoking status	Never	248 965	1021	207	88	56
	Former	187 670	1249	321	150	65
	Present	241 125	3264	757	310	171
Pre-existing morbidity[a] at baseline	No	522 176	3659	618	231	156
	Yes	155 585	1875	667	317	136

[a] Myocardial infarction, angina pectoris, stroke, hypertension, hypercholesterolemia or diabetes mellitus
