# Supplementary material for: Traffic air pollution and mortality from cardiovascular disease and all causes: a Danish cohort study
Source: Environ Health. 2012 Sep 5;11:60. doi: 10.1186/1476-069X-11-60 (PMC3515423; doi:10.1186/1476-069X-11-60)
Supplement: Additional file 5 — Table S4. Mortality rate ratios in association with non-logged time-weighted average concentration of NO2 from 1971 onwards at residential addresses. [file 1476-069X-11-60-S5.pdf]

Table S4 Mortality rate ratios in association with non-logged time-weighted average concentration of NO_2 from 1971 onwards at residential addresses

Mortality (ICD-10 codes)	N_{deaths}	Mortality rate ratio[a] (95% CI) per 10 μg/m^3 NO_2
All cause (except external: S-Z)	5534	1.08 (1.01-1.14)
Cardiovascular (I00-99)	1285	1.16 (1.03-1.31)
Ischemic heart disease (I20-25)	548	1.08 (0.89-1.30)
Cerebrovascular (I60-69)	292	1.09 (0.83-1.42)

The results were based on 677 761 person-years at risk among 52 061 cohort participants from baseline (1993-1997) through 2009

[a] Adjusted for sex, age (age was the time scale), calendar year, employment status, school attendance, occupation with potential for exposure to smoke and fumes, smoking status, smoking intensity, smoking duration, environmental tobacco smoke, alcohol, fat, fish, fruit and vegetables, fiber, folate, body mass index, waist circumference, physical active with sport, hormone replacement therapy, average gross income of municipality of residence in 1995 and road traffic noise at the baseline address. The Cox model stratified for marital status.
