# Supplementary material for: Traffic air pollution and mortality from cardiovascular disease and all causes: a Danish cohort study
Source: Environ Health. 2012 Sep 5;11:60. doi: 10.1186/1476-069X-11-60 (PMC3515423; doi:10.1186/1476-069X-11-60)
Supplement: Additional file 6 — Table S5. Mortality rate ratios in association with time-weighted average concentration of NO2 from 1971 onwards at residential addresses estimated in frailty models with municipality as a random effect. [file 1476-069X-11-60-S6.pdf]

Table S5. Mortality rate ratios in association with time-weighted average concentration of NO_2 from 1971 onwards at residential addresses estimated in frailty models with municipality as a random effect

Mortality (ICD-10 codes)	N_{deaths}	Mortality rate ratio[a] (95% CI)	p-value[b]
All cause (except external: S-Z)	5534	1.08 (0.98-1.18)	0.06
Cardiovascular (I00-99)	1285	1.27 (1.06-1.51)	> 0.50
Ischemic heart disease (I20-25)	548	1.12 (0.85-1.47)	> 0.50
Cerebrovascular (I60-69)	292	1.11 (0.76-1.63)	> 0.50

The results were based on 677 761 person-years at risk among 52 061 cohort participants from baseline (1993-1997) through 2009

[a] Given per doubling of the NO_2 concentration. Adjusted for sex, age (age was the time scale), calendar year, employment status, school attendance, occupation with potential for exposure to smoke and fumes, smoking status, smoking intensity, smoking duration, environmental tobacco smoke, alcohol, fat, fish, fruit and vegetables, fiber, folate, body mass index, waist circumference, physical active with sport, hormone replacement therapy, marital status, average gross income of municipality of residence in 1995, road traffic noise at the baseline address and municipality at baseline as a random effect.

[b] P-value for better fit of the frailty model compared to the standard model
