# Supplementary material for: Traffic air pollution and mortality from cardiovascular disease and all causes: a Danish cohort study
Source: Environ Health. 2012 Sep 5;11:60. doi: 10.1186/1476-069X-11-60 (PMC3515423; doi:10.1186/1476-069X-11-60)
Supplement: Additional file 7 — Table S6. Mortality rate ratios associated with exposure measures at residential addresses, based on cohort participants who lived at the same address from baseline (1993–1997) through 2009 and without adjustment for road traffic noise. [file 1476-069X-11-60-S7.pdf]

Table S6. Mortality rate ratios associated with exposure measures at residential addresses, based on cohort participants who lived at the same address from baseline (1993–1997) through 2009 and without adjustment for road traffic noise.

| Exposure | Mortality rate ratio[a] (95% confidence interval) | |
|---|---|---|
| | All causes (n=5182) | Cardiovascular disease (n=1213) |
| $NO_2$ (1-year mean) at address at baseline[b] | 1.15 (1.08-1.22) | 1.28 (1.12-1.45) |
| Major road within 50 m of address at baseline | 1.05 (0.96-1.15) | 1.10 (0.91-1.32) |
| Traffic load within 200 m of address at baseline[c] | 1.02 (1.01-1.04) | 1.05 (1.01-1.08) |

Results based on 569 637 person-years at risk for 43 678 cohort participants who lived at the same address from baseline (1993–1997) through 2009. This table is comparable with Table S2, but without adjustment for road traffic noise (post-hoc analysis).

[a] Adjusted for sex, age (age was the time scale), calendar year, employment status, school attendance, occupation with potential exposure to smoke and fumes, smoking status, smoking intensity, smoking duration, environmental tobacco smoking, alcohol, fat, fish, fruit and vegetables, fiber, folate, body mass index, waist circumference, physical activity with sport, hormone replacement therapy and average gross income of municipality of residence in 1995. The Cox model stratified for marital status.

[b] The mortality rate ratio is given per doubling of the $NO_2$ concentration.

[c] The mortality rate ratio is given per doubling of the traffic load
